# Imaging findings of granulocyte colony-stimulating factor-producing tumors: a case series and review of the literature

**DOI:** 10.1007/s11604-021-01130-8

**Published:** 2021-05-22

**Authors:** Shigeshi Kohno, Akihiro Furuta, Shigeki Arizono, Koji Tokunaga, Sei Nakao, Masahiro Tanabe, Tatsuki R. Kataoka, Hiroyoshi Isoda, Kaori Togashi

**Affiliations:** 1 Department of Diagnostic Imaging and Nuclear Medicine, Kyoto University Graduate School of Medicine, 54 Kawahara-cho, Shogoin, Sakyoku, Kyoto, 606-8507 Japan; 2 Department of Diagnostic Radiology, Kobe City Medical Center General Hospital, 2-1-1 Minatojimaminamimachi, Chuo-ku, Kobe, Hyogo 650-0047 Japan; 3 Department of Radiology, Osaka Red Cross Hospital, 5-30 Fudegasaki-cho, Tennoji-ku, Osaka, 543-8555 Japan; 4 Department of Radiology, Wakayama Red Cross Hospital, 4-20 Komatsubaradori, Wakayama, 640-8858 Japan; 5 Department of Radiology, Yamaguchi University Graduate School of Medicine, 1-1-1 Minamikogushi, Ube, Yamaguchi 755-8505 Japan; 6 Department of Diagnostic Pathology, Kyoto University Hospital, 54 Kawahara-cho, Shogoin, Sakyoku, Kyoto, 606-8507 Japan

**Keywords:** Granulocyte colony-stimulating factor-producing tumors, Imaging findings, Literature review, Magnetic resonance imaging, Positron emission tomography

## Abstract

Granulocyte colony-stimulating factor (G-CSF)-producing tumors have an aggressive clinical course. Here, we report five cases of G-CSF-producing tumors and review the literature, focusing on imaging findings related to tumor-produced G-CSF. In addition to our cases, we identified 30 previous reports of G-CSF-producing tumors on which ^18^F-fluorodeoxyglucose positron emission tomography (FDG-PET)/CT, bone scintigraphy, or evaluation of bone marrow MR findings was performed. White blood cell count, serum C-reactive protein, and serum interleukin-6 were elevated in all cases for which these parameters were measured. G-CSF-producing tumors presented large necrotic masses (mean diameter 83.2 mm, range 17–195 mm) with marked FDG uptake (mean maximum standardized uptake value: 20.09). Diffuse FDG uptake into the bone marrow was shown in 28 of the 31 cases in which FDG-PET/CT was performed. The signal intensity of bone marrow suggested marrow reconversion in all seven MRI-assessable cases. Bone scintigraphy demonstrated no significant uptake, except in two cases with bone metastases. Splenic FDG uptake was increased in 8 of 10 cases in which it was evaluated. These imaging findings may reflect the effects of tumor-produced G-CSF. The presence of G-CSF-producing tumors should be considered in patients with cancer who show these imaging findings and marked inflammatory features of unknown origin.

## Introduction

Granulocyte colony-stimulating factor (G-CSF) is a glycoprotein that stimulates the proliferation and differentiation of neutrophil progenitor cells in bone marrow [1]. There have been many reported cases of G-CSF-producing tumors in various organs, particularly the lung [2–5]. G-CSF-producing tumors generally exhibit significant hyperplastic and metastatic properties and have a poor prognosis [2–4]. Leukocytosis and inflammatory reactions due to the produced G-CSF and other inflammatory cytokines by tumors can be mistaken for other diseases, such as infections [2, 3, 5]. Early accurate diagnosis of G-CSF-producing tumors is important for determining treatment strategies and improving prognosis [6]. Examining the imaging features of G-CSF-producing tumors could aid clinicians in arriving at early diagnosis. To our knowledge, there have been no literature reviews related to imaging findings on G-CSF-producing tumors. In several previously reported cases of G-CSF-producing tumors, radiological examinations showed large necrotic masses [2, 3, 5]. However, the findings of large size and necrosis may also be present in less differentiated carcinomas and sarcomas, regardless of the tumors’ G-CSF-producing status. The possibility of G-CSF-producing tumors should be considered in conjunction with other radiological findings associated with tumor-producing G-CSF. Several previous studies have reported imaging findings related to tumor-produced G-CSF, such as diffuse ^18^F-fluorodeoxyglucose (FDG) uptake of bone marrow on FDG positron emission tomography (PET)/CT [7]. We speculated that imaging findings related to tumor-produced G-CSF would be similar even if the primary lesion was different. Therefore, we report five cases of G-CSF-producing tumors and review the related literature, focusing on imaging findings related to tumor-produced G-CSF.

## Presentation of cases

### Case 1

**Fig. 1:**
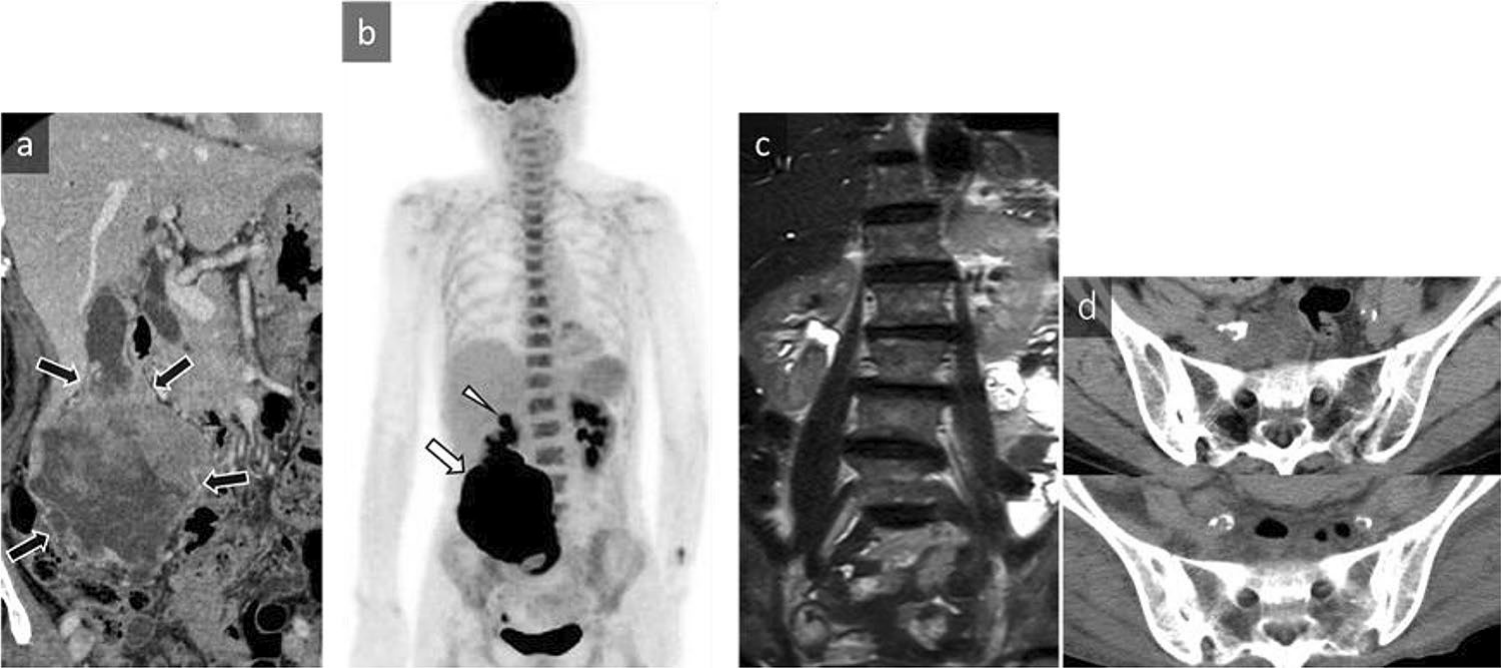
A 79-year-old woman with G-CSF-producing gallbladder carcinoma. **a** Coronal contrast-enhanced CT shows a 9.5-cm exophytic mass in the fundus of the gallbladder with geographic hypoattenuation and enhancement in the peripheral component (black arrow). **b** An ^18^F-FDG-PET/CT maximum intensity projection image demonstrates marked FDG uptake in the gallbladder tumor (maximum standardized uptake value = 48.7, white arrow) and gallbladder neck lymph node (white arrowhead), with diffuse uptake in the bone marrow of the spine and pelvis. The splenic uptake appears more intense than the hepatic uptake. **c** The signal intensity of bone marrow on T2-weighted (half Fourier acquisition single shot turbo spin echo [HASTE]) coronal images is lower than that of an age-matched healthy woman. **d** Axial CT scans of bone marrow in the spine and pelvic regions on admission (top panel, serum white blood cell count: 15,200) and 3 months after admission (bottom panel, serum white blood cell count: 59,800). CT attenuation of the bone marrow increased in parallel with tumor progression and elevated serum WBC count

A 79-year-old woman with prolonged fever and right lower abdominal pain was admitted to our hospital for investigation of a right abdominal mass. Laboratory tests performed at admission showed leukocytosis [white blood cell (WBC) count, 15,200/µL; neutrophils, 87%] and an elevated serum level of C-reactive protein (CRP, 7.5 mg/dL). Blood culture showed no growth. Contrast-enhanced CT showed a 9.5 cm exophytic mass in the fundus of the gallbladder with geographic hypoattenuation and enhancement in the peripheral component (Fig. 1a). FDG-PET/CT demonstrated marked FDG uptake in the gallbladder tumor and gallbladder neck lymph node, with diffuse uptake in the bone marrow and increased uptake in the spleen (Fig. 1b). MRI showed that the signal intensity of bone marrow on T2-weighted images (T2WI) was lower than that of an age-matched healthy woman (Fig. 1c). A biopsy specimen of the tumor revealed undifferentiated carcinoma. Although no infectious foci were detected, administration of antibiotics was continued from hospitalization because of prolonged fever of unknown origin. The patient was treated using a combination of gemcitabine and cisplatin chemotherapy. A decrease in tumor size and improved leukocytosis were temporarily observed after chemotherapy. However, the patient’s general condition became too deteriorated to continue chemotherapy. CT attenuation of bone marrow in the spine and pelvic regions changed in parallel with tumor progression and serum WBC count (Fig. 1d). The patient died 3 months after admission because of tumor progression. Immunostaining at autopsy revealed that the tumor cells were positive for G-CSF. Furthermore, the systemic bone marrow showed hyperplasia, indicating elevated bone marrow activity. Further immunostaining of the initial biopsy also revealed that the tumor cells were positive for G-CSF, confirming that the tumor cells produced G-CSF.

### Case 2

A 67-year-old woman with knee osteoarthritis, prolonged fever, and leukocytosis was treated with total knee arthroplasty. Microscopic examination of the synovium and synovial fluid revealed chronic synovitis with prominent neutrophils. Bacterial culture of the synovial fluid was negative. She was treated with antibiotics for the inflammatory features of unknown origin; however, her fever and elevated inflammatory reaction continued during postoperative follow-up. Contrast-enhanced CT showed a 7.9 cm exophytic mass that extended from the gallbladder to the liver, with geographic hypoattenuation suggesting necrosis, 3 months after surgery. FDG-PET/CT demonstrated FDG uptake in the gallbladder tumor, lymph nodes, and liver metastasis, with diffuse uptake in the bone marrow and increased uptake in the spleen. T2-weighted MRI showed that the signal intensity of bone marrow was lower than that of an age-matched healthy woman. Laboratory tests showed marked leukocytosis (WBC count, 33,000/µL; neutrophils, 89%) and elevated CRP (9.2 mg/dL) and serum G-CSF (214 pg/mL) levels. No infectious foci were detected, and blood culture showed no growth. A biopsy of the tumor revealed undifferentiated carcinoma. Bone marrow biopsy showed no neoplastic disease. The patient was treated with combined gemcitabine and cisplatin chemotherapy. After chemotherapy, the tumor size decreased, and improvements in leukocytosis and tumor markers were observed. CT attenuation of bone marrow in the spine and pelvic regions was decreased in parallel with tumor regression and serum WBC count. Chemotherapy was discontinued because of renal dysfunction and interstitial pneumonia, and the patient died of tumor progression 10 months after admission. Additional immunostaining revealed that tumor cells in the initial biopsy were positive for G-CSF.

### Case 3

**Fig. 2:**
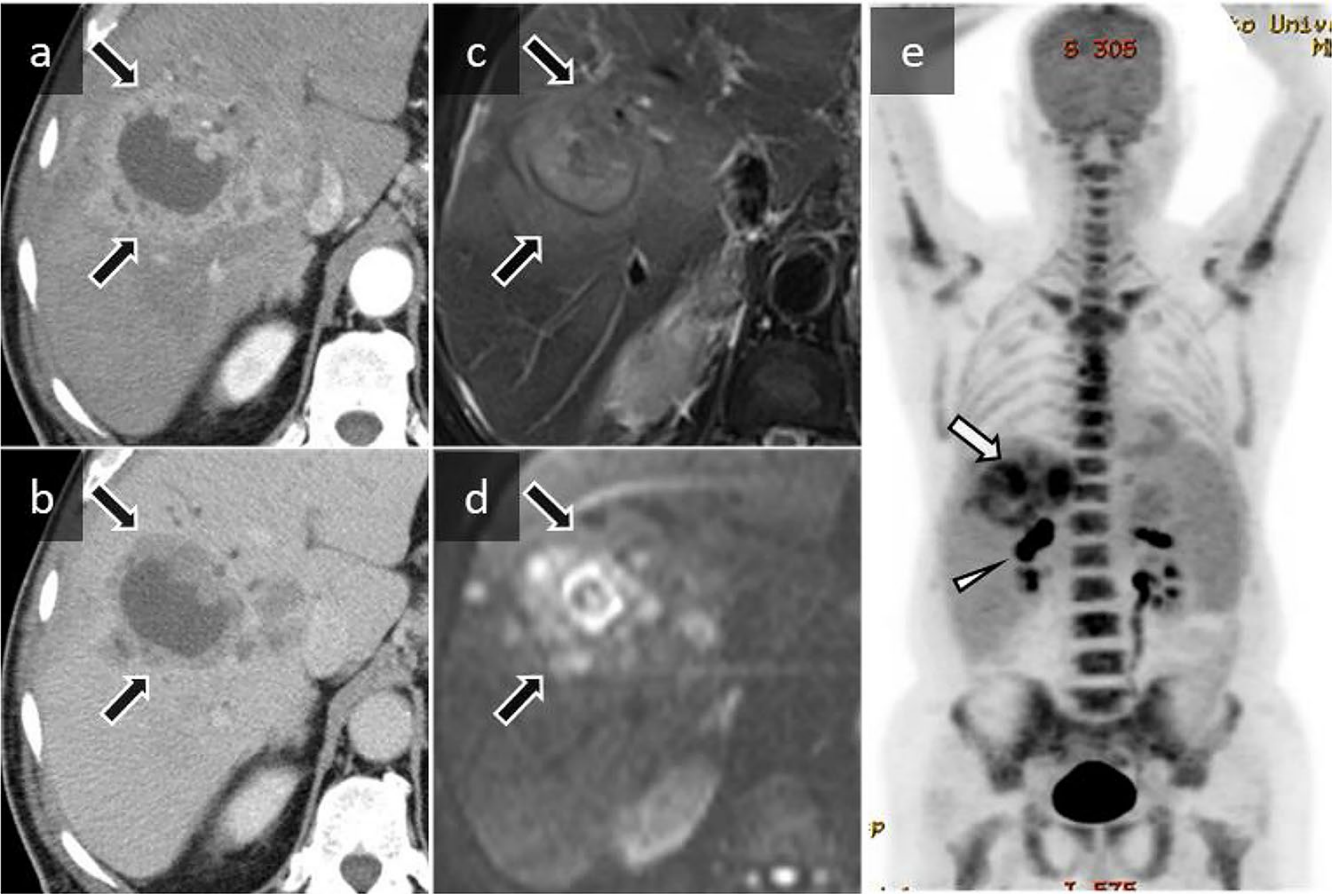
A 62-year-old man with G-CSF-producing hepatocellular carcinoma. **a, b** Axial contrast-enhanced CT image shows multiple rounded masses in the right lobe of the liver with geographic hypoattenuation and enhancement in the peripheral component, which are enhanced in the early phase and washed out in the late phase (black arrows). **c, d** Axial fat-suppressed turbo spin echo T2-weighted and diffusion-weighted imaging at b = 1000 s/mm^2^ shows that the signal intensity of the masses is mild hyperintensity relative to liver. **e** An ^18^F-FDG-PET/CT maximum intensity projection image demonstrates FDG uptake in the hepatic tumor (maximum standardized uptake value = 12.2, white arrow) and lymph node metastasis (white arrowhead) and diffuse uptake in the bone marrow of the spine, pelvis, and proximal metaphysis of the long bones. The splenic uptake is slightly more intense than the hepatic uptake

A 62-year-old man with a history of clearance of hepatitis C virus using interferon therapy was admitted to his local hospital for prolonged fever and weight loss. Radiological examinations showed multiple hepatic lesions. He was treated with antibiotics after an initial diagnosis of cholecystitis and liver abscess; however, his symptoms did not improve over the next 4 months. A biopsy of the hepatic lesion was performed, which revealed hepatocellular carcinoma. He was referred to our hospital for diagnosis and to determine treatment strategies. Laboratory tests at admission showed marked leukocytosis (WBC count, 24,400/µL; neutrophils, 84%) and elevated CRP (13.0 mg/dL) and serum G-CSF (549 pg/mL) levels. No infectious foci were detected, and blood cultures showed no growth. Contrast-enhanced CT taken at our hospital showed multiple masses in the liver with geographic hypoattenuation and enhancement in the peripheral component (Fig. 2a, b). MRI showed that the tumor had mild hyperintensity on T2WI and diffusion-weighted imaging (DWI) at b = 1000 s/mm^2^ (Fig. 2c, d). FDG-PET/CT demonstrated FDG uptake in the hepatic tumor and lymph node metastasis, with diffuse uptake in the bone marrow and increased uptake in the spleen (Fig. 2e). The tumor biopsy revealed poorly differentiated hepatocellular carcinoma with marked neutrophil infiltration, which was suspected to be a G-CSF-producing tumor.

### Case 4

A 70-year-old man with a history of chronic hepatitis C virus was admitted to our hospital with prolonged fever. Laboratory tests performed at admission showed marked leukocytosis (WBC count, 34,900/µL; neutrophils, 97%) and elevated levels of CRP (8.4 mg/dL), G-CSF (452 pg/mL), and interleukin-6 (IL-6; 81.4 pg/mL). No infectious foci were detected, and blood culture showed no growth. Contrast-enhanced CT showed a round mass of 2.7-cm in the liver with geographic hypoattenuation and enhancement in the peripheral component, and multiple lymph node metastases. MRI showed that the tumor was mildly hyperintense on T2WI and DWI at b = 1000 s/mm^2^. Bone scintigraphy showed no significant uptake in the spine, whereas T2-weighted MRI showed that the signal intensity of bone marrow was lower than normal. Microscopic examination of the liver tumor revealed undifferentiated carcinoma. The patient was treated with sorafenib, and improved leukocytosis was temporarily observed; however, the tumor increased in size, his general condition rapidly worsened, and he died 5 months after admission.

### Case 5

**Fig. 3:**
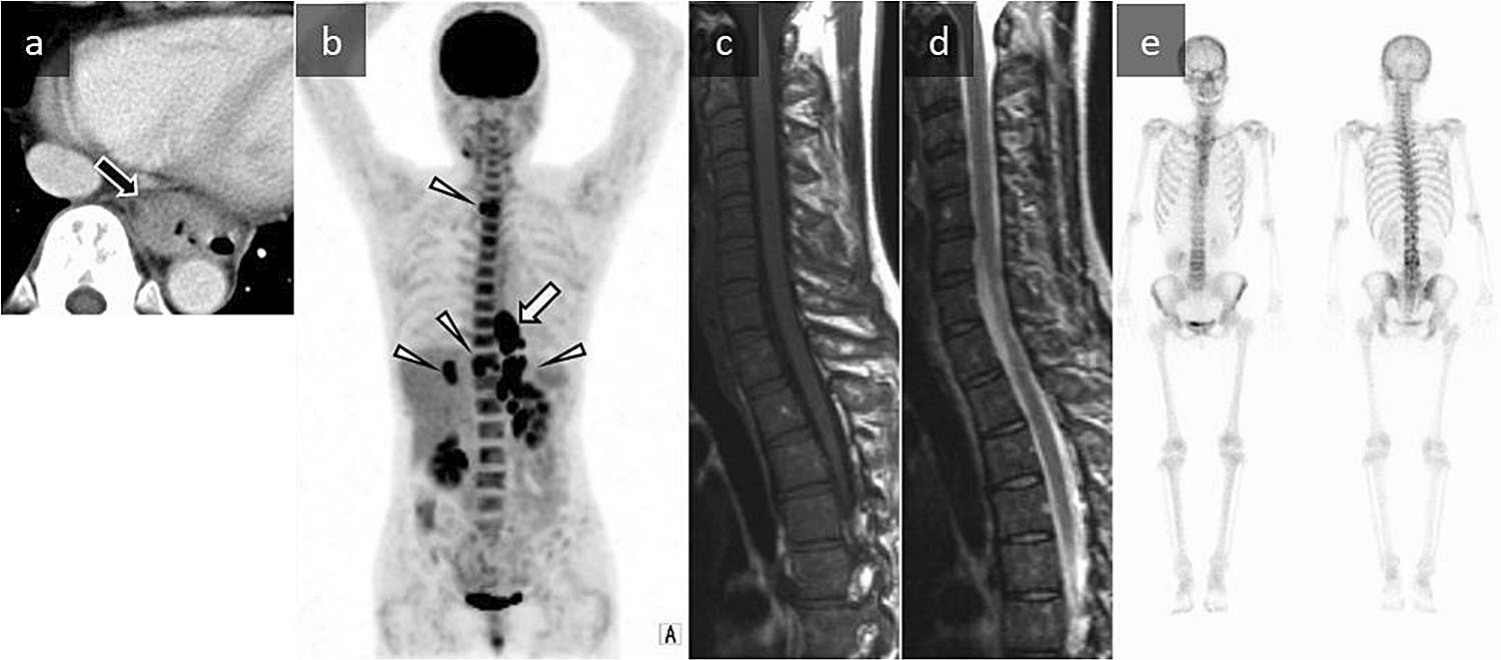
A 52-year-old woman with G-CSF-producing carcinoma of the esophagus. **a** Axial contrast-enhanced CT shows a well-defined and nonnecrotic mass 4.6 cm in diameter located in the esophagus (black arrow). **b** An ^18^F- FDG-PET/CT maximum intensity projection image demonstrates marked FDG uptake in the esophageal tumor (maximum standardized uptake value = 32.4, white arrow), lymph nodes, and liver metastases (white arrowheads) with diffuse uptake in the bone marrow of the spine. The splenic uptake is slightly more intense than the hepatic uptake. **c, d** Spinal sagittal MRI shows that the signal intensity of the bone marrow is lower than that of an age-matched healthy woman on spin echo T1- and turbo spin echo T2-weighted imaging. **e** Bone scintigraphy shows no significant uptake in the spine

A 52-year-old woman was admitted to our hospital for investigation of difficulty swallowing. Upper gastrointestinal endoscopy revealed a tumor in the lower thoracic esophagus. Laboratory tests performed at admission showed leukocytosis (WBCs, 24,570/µL; neutrophils, 88.5%) and an elevated CRP level (1.74 mg/dL). Contrast-enhanced CT showed a well-defined, nonnecrotic mass 4.6 cm in diameter located in the esophagus (Fig. 3a). FDG-PET/CT demonstrated marked FDG uptake in the esophageal tumor, lymph nodes, and liver metastases, with diffuse uptake in the bone marrow and increased uptake in the spleen (Fig. 3b). Spinal MRI showed that the signal intensity of the bone marrow was lower than that of an age-matched healthy woman on T1-weighted images (T1WI) and T2WI (Fig. 3c, d). Bone scintigraphy showed no significant uptake in the spine (Fig. 3e). A biopsy of the tumor showed poorly differentiated squamous cell carcinoma. Additional immunostaining was performed after referring to the observed diffuse FDG uptake of bone marrow and MR findings of bone marrow change, and the tumor cells were positive for G-CSF. Laboratory tests showed a high serum G-CSF level (568 pg/mL). The patient was treated with cisplatin and fluorouracil chemotherapy as the first-line regimen, followed by docetaxel and nedaplatin as the second-line regimen. After the first course of chemotherapy, the patient’s serum level of G-CSF decreased to 142 pg/mL, and the WBC count decreased to within normal ranges. However, the serum G-CSF level and WBC count were re-elevated after five courses of chemotherapy, and follow-up CT detected tumor regrowth and new metastatic lesions. The patient died 16 months after admission because of tumor progression.

## Literature review

A literature review using the PubMed database was performed focusing on imaging findings related to tumor-produced G-CSF, such as FDG uptake of tumors and bone marrow change. Published English articles were searched from 2000 to 2020 using the following search terms: “Granulocyte colony stimulating factor producing tumor” and “FDG”, or “Granulocyte colony stimulating factor producing tumor” and “hyperplasia bone marrow”, or “Granulocyte colony stimulating factor producing tumor” and “Bone scintigraphy”. After scrutinizing these papers and references, 28 cases were identified from publications and their references [6–32]. Additionally, we searched the Japan Medical Abstracts Society database looking for the keywords “G-CSF producing” and “Bone scintigraphy”, or “G-CSF producing” and “MRI” and “bone marrow”. Two additional cases were identified from these publications [33, 34]. Including our 5 cases, a total of 35 cases were reviewed.

### Clinical characteristics of G-CSF-producing tumors

The patients consisted of 26 men and 9 women with a mean age of 62.6 years (range 31–79 years). Although the patients had a wide variety of chief complaints because of their different primary sites, fever was reported in 21 of the 35 cases. In our cases 1–4, prolonged fever was a chief complaint, and it may have been caused by inflammatory cytokines produced by the tumor. Laboratory tests showed marked leukocytosis (mean WBC count 30,239/µL, range 14,080–79,600/µL) and elevated serum CRP levels (mean 11.91 mg/dL, range 0.99–27.43 mg/dL) in all patients described. However, no infectious foci were described in any of the patients and production of inflammatory cytokines by the tumors was suggested. Previous studies have also reported that G-CSF-producing tumors produce other inflammatory cytokines, such as IL-6 and IL-8 [11, 35]. Serum IL-6 levels (mean 141.3 pg/mL, range 14.6–727 pg/mL) were elevated in all 10 patients in whom the level was measured, and this can also explain the inflammatory reactions. Serum G-CSF levels (mean 326 pg/mL, range 28–2670 pg/mL) were elevated in all 32 patients described. Serum G-CSF is not usually measured unless G-CSF-producing tumors are suspected by clinicians, as in our case 1, which was not diagnosed until autopsy. The possibility of G-CSF-producing tumors should be considered in patients with cancer who have marked leukocytosis and inflammatory reactions of unknown origin. Serum G-CSF levels and WBC counts were correlated with tumor progression and treatment response in all 26 cases in which the trajectories of hematologic data were reported. These values can serve as indicators of therapeutic effect and recurrence in patients diagnosed with G-CSF-producing tumors [36]. G-CSF-producing tumors generally have a poor prognosis. In 24 cases with reported patient outcomes, over 12 months from the beginning of therapy, 16 patients died and only 8 survived.

### Imaging findings

Imaging findings of our five cases are summarized in Table 1.

**Table 1:** Imaging findings of our five cases with G-CSF-producing tumors

	Case 1	Case 2	Case 3	Case 4	Case 5
Primary tumor	Gallbladder	Gallbladder	Liver	Liver	Esophagus
Tumor size (mm)	95	79	63	27	46
Findings suggestive of necrosis	+	+	+	+	−
SUV_max_	48.7	8.8	12.2	NA	32.4
Bone marrow					
Diffuse FDG uptake	+	+	+	NA	+
FDG uptake site	Spine and pelvis	Spine, pelvis, and proximal metaphysis of long bones	Spine, pelvis, and proximal metaphysis of long bones	NA	Spine
Signal intensity compared with an age-matched healthy case (T2WI/T1WI)	Lower/NA	Lower/NA	Lower/NA	Lower/NA	Lower/lower
Bone scintigraphy	NA	NA	NA	Not significant	Not significant
Increased FDG uptake in spleen	+	+	+	NA	+
Metastasis	+	+	+	+	+

*G-CSF* granulocyte colony-stimulating factor, *FDG* ^18^F-fluorodeoxyglucose, *SUV* standardized uptake value, *T2WI* T2-weighted imaging, *T1WI* T1-weighted imaging, *NA* not available

### Primary tumors

Imaging findings of the primary tumors in the literature are summarized in Table 2. G-CSF-producing tumors were found in various organs, with lung lesions being the most common. Because we mainly extracted patients with G-CSF-producing tumors who underwent FDG-PET/CT, on which it is sometimes difficult to detect urologic tumors, there were no cases of urinary tract or kidney tumors in this literature review [37]. A previous study reviewed the primary site of 420 cases of G-CSF-producing tumors in the PubMed and Japan Medical Abstracts Society databases: 94 cases in lung (22.4%); 57 cases in urinary tract (13.6%); 38 cases in stomach and duodenum (9%); 30 cases in esophagus (7.1%); 23 cases in liver (5.5%); 20 cases in pancreas (4.8%); 20 cases in uterus (4.8%); 16 cases in biliary tract and gallbladder (3.8%); 15 cases in small intestine and colon (3.6%); 14 cases in thyroid (3.3%); 13 cases in kidney (3.1%); 12 cases in ovary (2.9%); 10 cases in pleura (2.4%); 10 cases in oral cavity (2.4%); 4 cases in breast (0.95%); 4 cases in peritoneum and mesentery (0.95%); and 32 cases in other areas (7.6%) [29]. G-CSF-producing tumors can occur in various organs, especially the lung.

**Table 2:** Imaging findings of the primary tumor in patients with G-CSF-producing tumors (*n* = 30)

	Age	Sex	Primary site	Size (mm)	Findings suggestive of necrosis	SUV_max_ (primary site)	Histopathology
Morooka et al. [7]	67	M	Lung	NA	NA	13.9	Spindle cell carcinoma
Morooka et al. [7]	64	M	Lung	60	NA	26.4	Pleomorphic carcinoma
Takahashi et al. [8]	74	M	Lung	NA	NA	NA	Pleomorphic carcinoma
Tsutsumi et al. [9]	58	M	Lung	17	NA	6.12	Adenocarcinoma
Yoshinaga et al. [10]	73	M	Lung	90	NA	NA	Poorly differentiated adenocarcinoma
Matsumoto et al. [11]	52	M	Lung	40	NA	NA	Pleomorphic carcinoma
Kaira et al. [12]	73	M	Lung	NA	NA	NA	Poorly differentiated carcinoma
Hidaka et al. [13]	57	M	Lung	60	NA	25.79	Pleomorphic carcinoma
Takagi et al. [14]	47	M	Lung	96	NA	7.06	Poorly differentiated adenocarcinoma
Makino et al. [15]	66	M	Lung	60	NA	NA	Pleomorphic carcinoma
Kawaguchi et al. [16]	62	M	Stomach	82	NA	NA	Poorly differentiated adenocarcinoma
Tsuruta et al. [17]	67	M	Stomach	NA	NA	NA	Neuroendocrine carcinoma
Kohno et al. [18]	46	M	Liver	100	+	25	Poorly differentiated hepatocellular carcinoma
Suzumura et al. [19]	61	F	Bile duct	150	+	NA	Poorly differentiated adenocarcinoma
Shimamoto et al. [20]	58	F	Tongue	40	NA	NA	Poorly differentiated squamous cell carcinoma
Kuroshima et al. [21]	78	M	Tongue	42	+	22.19	Squamous cell carcinoma
Zweifel et al. [22]	57	M	Thyroid	NA	NA	NA	Anaplastic carcinoma
Fukui et al. [23]	56	F	Breast	101	+	NA	Metaplastic breast carcinoma
Oshikiri et al. [6]	65	M	Esophagus	195	NA	NA	Well-differentiated squamous cell carcinoma
Suzumura et al. [24]	78	M	Gallbladder	120	+	16.89	Adenosquamous cell carcinoma
Kitade et al. [25]	68	M	Pancreas	72	+	17.1	Anaplastic carcinoma
Koyama et al. [26]	31	F	Uterus	NA	NA	16.7	Squamous cell carcinoma
Kobara et al. [27]	39	F	Ovary	60	NA	NA	Squamous cell carcinoma
Hara. [28]	63	M	Retroperitoneum	150	NA	18.5	Dedifferentiated liposarcoma
Yasui et al. [29]	75	M	Occult primary	NA	+	NA	Adenocarcinoma
Fujiwara et al. [30]	76	M	Pleura	110	NA	18.7	Malignant pleural mesothelioma
Kazama et al. [31]	50	M	Colon	72	NA	25.12	Undifferentiated pleomorphic sarcoma
Matsuyama et al. [32]	69	M	Bile duct	NA	+	NA	Poorly differentiated adenocarcinoma
Nakade et al. [33]	60	F	Ovary	100	NA	NA	Carcinosarcoma
Hondo et al. [34]	NA	M	Esophagus	120	NA	NA	Poorly differentiated squamous cell carcinoma

*M* male, *F* female, *FDG-PET* ^18^F-fluorodeoxyglucose positron emission tomography, *G-CSF* granulocyte colony-stimulating factor, *SUV* standardized uptake value, *MIP* maximum intensity projection, *NA* not available

The tumor size was relatively large (mean diameter 83.2 mm, range 17–195 mm), suggesting that they were aggressive. Hypoattenuation suggesting necrosis was shown in 12 of the 13 cases for which imaging findings of the tumors were described. The primary lesions in our cases 1–4 presented with extensive necrosis (geographic necrosis) (Figs. 1a, 2a). These findings may reflect the extensive necrosis that is frequently seen pathologically in G-CSF-producing tumors [38]. The finding of large masses with geographic necrosis may aid in the diagnosis of G-CSF-producing tumors. However, no studies have evaluated the pattern of necrosis in G-CSF-producing tumors, and further research is needed in this respect. There are many case reports of G-CSF-producing tumors initially treated with antibiotics because of misdiagnosis as abscesses [5, 39–46]. In our cases 3 and 4, the imaging findings were very similar to liver abscess (Fig. 2b). Moreover, our case 3 was initially diagnosed with liver abscess, and delays in the patient’s diagnosis led to delayed tumor treatment.

MR findings of these types of tumors have not been described well in the literature. In our cases 3 and 4, the geographic necrosis (which showed very similar findings to liver abscess on CT) was mildly hyperintense on T2WI and DWI at b = 1000 s/mm^2^ (Fig. 2c, d). Because abscesses are often very hyperintense on T2WI and DWI, MRI might help to distinguish between an abscess and tumor necrosis [47, 48].

FDG-PET/CT demonstrated marked FDG uptake by the tumors (mean maximum standardized uptake value (SUV_max_) 20.09, range 6.1–48.7) (Figs. 1b, 2e, 3b). This marked uptake by tumors could be related to the tumors’ large size, higher grade of malignancy, and marked inflammatory cell infiltration [7]. G-CSF-producing tumors are thought to be more common in less differentiated carcinomas [2–4]. The pathological findings of our cases and literature review also included 11 cases of poorly differentiated carcinoma and 4 cases of undifferentiated carcinoma or sarcoma. In addition, G-CSF induces marked activation of granulocytes. Tumors showing inflammatory cell infiltration, such as granulocytes and granulation tissue, are known to show elevated glucose metabolism. [49].

G-CSF-producing tumors often presented as large necrotic masses with marked FDG uptake in our cases and the literature. These findings with high SUV_max_ values may suggest that the tumors produce G-CSF, but they should be differentiated in conjunction with clinical and other radiological findings associated with tumor-producing G-CSF, such as marked inflammatory features and bone marrow change.

### Bone marrow

There are many case reports of G-CSF-producing tumors with bone marrow change on radiological examinations, such as diffusely increased FDG uptake of the bone marrow on FDG-PET/CT and bone marrow reconversion on MRI [6–33]. Bone marrow change after G-CSF administration is known as hyperplasia of the red marrow, which indicates hyperactive bone marrow [50]. FDG-PET/CT demonstrated diffusely increased FDG uptake of the bone marrow in 28 of 31 cases. However, diffuse FDG uptake of the bone marrow could be present in long-standing severe anemia, post-chemotherapy, diffuse bone metastases, malignant lymphoma, and myeloproliferative diseases such as leukemia [51]. Therefore, it is necessary to confirm the presence of the primary tumor and the patient’s medical history and blood data to differentiate the causes of diffuse FDG uptake in the bone marrow. In particular, marked FDG uptake in the bone marrow in patients with cancer requires differentiation from bone metastasis. Bone metastases normally show a multifocal and inhomogeneous distribution of FDG uptake [52]. The clinical diagnosis of the bones in our literature review was G-CSF-stimulated bone marrow in all cases, as it had marked inflammatory features of unknown origin and uniform distribution of the FDG uptake. In the 11 cases in which histological assessment was performed, the histopathology of these bones was bone marrow hyperplasia in 9 cases and no signs of neoplastic disease in 2 cases. Diffuse FDG uptake in bone marrow may reflect increased metabolism and cellularity of red bone marrow in response to tumor-produced G-CSF [1]. The pattern of diffuse FDG uptake in the G-CSF-stimulated bone marrow was symmetrically distributed, and such bone marrow was primarily located in the spine, pelvis, and proximal metaphysis of the long bones, especially heavily distributed in the spine in all of our cases and the literature (Figs. 1b, 2e, 3b). Marrow reconversion from yellow to red marrow can occur in response to different physiologic stimuli, and it has been reported in patients receiving chemotherapeutic regimens and G-CSF to reduce the associated bone marrow suppression [50]. Marrow reconversion is the reverse process of the natural replacement of red marrow by yellow marrow, and the process of age-related bone marrow conversion eventually prevails in the spine, pelvis, and proximal metaphysis of long bones [53]. Therefore, diffuse FDG uptake due to marrow reconversion may be distributed in these bones.

On MRI, it is possible to evaluate the signal intensities of bone marrow. On T1WI and T2WI, red marrow has a lower signal than that of yellow marrow because of its differences in water and fat content [50]. In two previously reported cases, G-CSF-producing malignant ovarian tumors were suspected because of marked leukocytosis and MR findings of pelvic bone intensity, suggesting marrow reconversion [27, 33]. In our five cases and previous literature cases that assessed the signal intensities of bone marrow (total: seven cases), the signal intensity in the spine and pelvic regions was lower than that in age-matched healthy cases on T2WI and T1WI (Figs. 1c, 3c, 3d). Such MR findings suggestive of marrow reconversion might aid in the diagnosis of G-CSF-producing tumors. MRI is also useful for differentiating benign from malignant bone marrow lesions [54, 55]. Although chemical shift imaging was not performed in our cases or those in the literature, a previous study reported that a signal drop loss of more than 20% in chemical shift imaging was useful to rule out malignant lesions [54].

Bone scintigraphy was performed in our two cases and five literature cases [8, 18, 28, 32, 34]. Bone scintigraphy only demonstrated abnormal uptake in the two cases with bone metastases, and there was no significant uptake in the other five cases diagnosed with G-CSF-stimulated bone marrow (Fig. 3b–e). A previous bone scintigraphy study reported no abnormal uptake in marrow reconversion and a significant difference in uptake between marrow reconversion and bone metastasis [55]. Bone scintigraphy may be useful to rule out bone metastasis in patients with G-CSF-producing tumors.

A previous study reported that the CT attenuation of focal hyperplasia in bone marrow was equal to or slightly higher than that of adjacent normal-appearing bone marrow [55]. In our cases 1 and 2, the CT attenuation of bone marrow without cortical bone and extensive calcification appeared to be higher than that of age-matched healthy cases and changed in parallel with tumor progression and serum WBC count. This may reflect change in bone marrow activity [Fig. 1d]. These bones’ histopathology showed hyperplasia of the bone marrow and no signs of neoplastic disease. The high CT attenuation of bone marrow, which reflects hyperplasia of red marrow, might aid with the diagnosis of tumor-produced G-CSF in patients with cancer who have marked inflammatory features of unknown origin. However, no studies have evaluated the CT attenuation of bone marrow after G-CSF administration, and further research is needed on this respect.

### Spleen

Increased FDG uptake in the spleen was described in 8 of the 10 cases in which splenic uptake was evaluated [7, 11, 20, 22, 26]. On FDG-PET/CT, splenic uptake is normally less than hepatic uptake [56, 57]. In all four of our cases in which FDG-PET/CT was performed, the splenic FDG uptake was diffusely more intense than the hepatic uptake (Figs. 1b, 2e, 3b). Increases in splenic diffuse FDG uptake have often been reported after G-CSF therapy, and the increased uptake in these cases may reflect increases in extramedullary granulopoiesis and erythropoiesis induced by tumor-produced G-CSF [58, 59]. Because increased splenic uptake may also be present in lymphoma, sarcoidosis, and many inflammatory or hematopoietic diseases, the possibility of G-CSF-producing tumors should be considered in conjunction with clinical and other radiological findings [60].

### Others

Tumor-produced G-CSF has been shown to affect tumor progression by facilitating tumor-associated angiogenesis and enhanced metastatic ability [61, 62]. In our literature review, CT and FDG-PET/CT demonstrated lymph node metastasis or distant metastasis before treatment in 18 of 35 cases, suggesting that the tumors had highly metastatic properties. Although this finding is nonspecific, G-CSF-producing tumors often present with metastases in their initial appearance.

Several cases of aortitis developing after G-CSF administration have been recently reported in the literature [63]. G-CSF-associated aortitis is extremely rare, with a frequency of 0.3–0.47% [64, 65]. CT and MRI show wall thickening of the aorta and its major branches, soft tissue attenuation surrounding these vessels, and other large vessel vasculitis [66]. Although the mechanism of G-CSF-associated aortitis is still unclear, previous studies have reported the association between G-CSF treatment and the development of aortitis [64]. However, no literature has reported aortitis appearing alongside G-CSF-producing tumors, and further research is needed.

## Conclusion

G-CSF-producing tumors should be considered in patients with cancer who have marked inflammatory features of unknown origin. Imaging findings including large necrotic masses, marked FDG uptake by the tumors, diffuse FDG uptake of the bone marrow, increased FDG uptake in the spleen, and MR findings of marrow reconversion may indicate tumor-produced G-CSF in such patients. MRI and bone scintigraphy may be useful for differentiating marrow reconversion from bone metastasis when FDG-PET/CT demonstrates diffuse FDG uptake in bone marrow.
